# Children’s Inequity Aversion in Procedural Justice Context: A Comparison of Advantageous and Disadvantageous Inequity

**DOI:** 10.3389/fpsyg.2017.01855

**Published:** 2017-10-18

**Authors:** Xiaoju Qiu, Jing Yu, Tingyu Li, Nanhua Cheng, Liqi Zhu

**Affiliations:** ^1^CAS Key Laboratory of Behavioral Science, Institute of Psychology, Chinese Academy of Sciences, Beijing, China; ^2^University of Chinese Academy of Sciences, Beijing, China; ^3^Eunice Kennedy Shriver National Institute of Child Health and Human Development, Bethesda, MD, United States

**Keywords:** knowledge-behavior gap, advantageous inequity aversion, disadvantageous inequity aversion, procedural justice, fairness concerns

## Abstract

There are two forms of unfairness widely studied in resource allocation settings: disadvantageous inequity (DI) in which one receives less than the partner and advantageous inequity (AI) in which one receives more than the other. We investigated children’s aversion to AI and DI in a procedural justice context. Children of 4-, 6-, and 8- years old were asked to spin a wheel (procedure) to decide how to allocate two different rewards with others. In each condition, they chose between a fair procedure providing equal chances for the two parties to get the bigger reward, and an unfair procedure (either a disadvantageous procedure in the DI condition, or an advantageous procedure in the AI condition). Results showed that children in the two younger age groups had a preference for the unfair procedure that would maximize their own profit in AI, but a greater aversion to the unfair procedure that would disadvantage them in DI. Eight-year-olds, however, had a greater preference for the fair procedure in AI than the 6-year-olds. In addition, the discrepancy between aversion to AI and DI disappeared in the 8-year-olds. The findings indicate children’s development of other-oriented concerns such as fairness concern and altruism in procedural justice, consistent with previous findings in distributive justice.

## Introduction

Fairness is crucial for maintaining interpersonal relationship and promoting social cooperation (Brosnan and de Waal, 2014; Tomasello, 2016). Fairness in resource allocation can be achieved in two ways: distributive justice and procedural justice (Grocke et al., 2015). Distributive justice often means equal amount of resources allocated to each individual, with other things (e.g., need, effort) being equal. When distributive justice is hard to achieve, fairness may be maintained through procedural justice — providing equal opportunities for each individual to get resources. Whereas young children even infants possess the expectation for fairness (Geraci and Surian, 2011; Sloane et al., 2012; Meristo and Surian, 2013), they tend to maximize self-interest in actual resource distributions until the age of 8 (Fehr et al., 2008; Kogut, 2012; Smith et al., 2013). However, much less is known about children’s sharing behavior in procedural justice context. The present study aims to find out whether the knowledge-behavior gap exists in the field of procedural justice by comparing children’s aversion to disadvantageous inequity (DI) and to advantageous inequity (AI).

Children develop the sense of fairness from early on. Ten-month-old infants expect resources to be allocated equally (Geraci and Surian, 2011; Sloane et al., 2012; Meristo and Surian, 2013). They stare longer at unequal distributions (Sloane et al., 2012), become more socially engaged with the fair distributor (Geraci and Surian, 2011), and expect the fair distributor to be rewarded (Meristo and Surian, 2013). Three-year-olds acknowledge that items should be evenly divided between themselves and the other recipients (Smith et al., 2013). Furthermore, they would respond negatively when they become a victim of an unfair distribution (Blake and McAuliffe, 2011; LoBue et al., 2011; Li et al., 2016). When observing a distribution made between other players, 6-year-old children would sacrifice some self-interest to punish the unequal distributor (McAuliffe et al., 2015). These findings suggest that children are aware of the fairness norm and are able to make social expectations based on their notion of fairness.

However, there is a knowledge-behavior gap (Blake et al., 2014). Children often fail to regulate their distributive behavior using the fairness norm before the age of 8. Children aged 3–4 would occupy most of the resources and feel satisfied in doing so when distributing resources between themselves and other recipients (Kogut, 2012; Smith et al., 2013). They showed no preference for the equal distribution (both got one item) if they can get a relative advantage by choosing the other choice (2,0—they got two and the other player got zero) (Fehr et al., 2008). The findings have been replicated in many other studies (Blake et al., 2015; Melis et al., 2015; Ulber et al., 2015; Cowell et al., 2016; Steinbeis, 2016). The knowledge-behavior gap is often represented as a discrepancy between DI aversion and AI aversion. Children at 3 have already shown strong aversion to DI favoring others in terms of negative emotional responses (LoBue et al., 2011), more willingness to sacrifice their own resources to prevent others from getting better reward (Takagishi et al., 2010; Sheskin et al., 2014), and a preference for equality in disadvantaged situations (Blake and McAuliffe, 2011; Li et al., 2016). In contrast, children of the same age are quite ready to accept or initiate unequal offers to maximize their relative interest (Blake and McAuliffe, 2011; LoBue et al., 2011; Sheskin et al., 2014). Only 8 out of 71 children aged 3–5 clearly rejected an advantageous offer (LoBue et al., 2011). By the age of 8, children start to show aversion to AI; they are able to allocate resources equally even their self-interest is involved, and are more satisfied in complying with the fairness norm than younger participants (Smith et al., 2013). In addition, children at 8 are willing to reject AI offers at a personal cost (Blake and McAuliffe, 2011; McAuliffe et al., 2013), and even disadvantage themselves by giving more resources to others to show their generosity (Shaw et al., 2016). Compared with children, adults are more tolerant of DI but rejecting AI at a higher degree when distributing resources (e.g., candies) that may not be very appealing to them (McAuliffe et al., 2014). The development of discrepancy between aversion to DI and to AI suggests that children are able to use their knowledge of fairness norm to secure their relative self-interest from early on, but not until later can they realize that the norm of fairness applies to others as well and show an aversion to AI (Brosnan and de Waal, 2014).

The discrepancy between aversion to DI and AI may be explained in three ways. First, different amount of cognitive control may be employed. Cognitive control is often associated with children’s inhibition of self-interest maximization and conforming to the norm of fairness (Steinbeis, 2016; Steinbeis and Over, 2017). As rejecting AI often requires a larger sacrifice and giving up a superior status compared to rejecting DI (e.g., losing 4 items rather than 1), one needs to make more cognitive effort to maintain fairness in AI (Brosnan and de Waal, 2014). For example, children aged 4–7 took a longer time in rejecting an advantageous offer than accepting it, whereas no such difference was found between rejecting and accepting DI offers (Blake and McAuliffe, 2011).

Because the discrepancy between aversion to DI and that to AI exists after controlling for the cost of rejecting the unequal offer (Williams and Moore, 2016) and in adults of several cultures (Blake et al., 2015), cognitive control alone cannot fully explain their difference. Another explanation is social comparison—an inclination to maximize one’s own welfare relative to others (Blake et al., 2014; Sheskin et al., 2014). Children may compare their resources with others’ (Blake and McAuliffe, 2011), and want to feel better or have more resources than others (Blake et al., 2014; Sheskin et al., 2014). This is especially true for children around 5 or 6, who are willing to take a cost to get more than others in resource distributions (Sheskin et al., 2014).

A third possible explanation for the discrepancy is that young children have not internalized the fairness norm (Kogut, 2012). Despite their awareness of the fairness norm, young children are unwilling to regulate their behavior with the fairness norm when there is no external force (Henrich et al., 2005; Kogut, 2012; Steinbeis et al., 2012; Smith et al., 2013). They may choose to satisfy their desire for more resources rather than obeying the norms (Kogut, 2012; Smith et al., 2013). As they grow older, children gradually learn to value the norm of fairness and actively use it to guide their behaviors (Kogut, 2012; Smith et al., 2013). The three explanations are equally plausible and may complement each other in explaining the knowledge-behavior gap.

However, most of the studies are on distributive justice. When equal distribution is impossible to achieve, fairness can be achieved by providing equal opportunities for each party to access the resource, that is, through procedural justice (Grocke et al., 2015). A fair procedure (e.g., a wheel) that provides each 50/50 chance to gain the bigger reward can substitute for equal outcomes (Bolton et al., 2005). Adults are also more likely to accept unequal outcomes based on a fair procedure rather than a biased procedure (Brockner and Wiesenfeld, 1996; Bolton et al., 2005). People value fair procedure not just because of the instrumental benefits it brings, but also due to its social implications — feelings of being respected and valued as an equal social member (Heuer and Stroessner, 2011).

Recently, studies have revealed that infants manifest probabilistic intuitions: they are sensitive to proportions and are capable of making the optimal choice based on probabilities (Téglás et al., 2007, 2011; Xu and Garcia, 2008; Denison et al., 2013; Denison and Xu, 2014). Furthermore, children can apply their knowledge of probability reasoning in procedural distribution and show a preference for procedural justice (Shaw and Olson, 2014; Grocke et al., 2015). Children at 8 understand that a fair procedure should entail equality of opportunities as well as randomness. They choose a wheel to distribute an extra item for another two recipients only when the wheel provides equal opportunities for both parties (Shaw and Olson, 2014). When self-interest is involved, children at 5 show a resistance of unequal outcomes coming out of an unfair procedure, but accept unequal outcomes from a fair procedure (Grocke et al., 2015). The results demonstrate that children concern about procedural justice as well. However, it remains unknown how children would maintain procedural justice when they can benefit from the distributive procedures.

Although people value procedural justice, they may become less concerned about the fairness of outcomes when using procedures to allocate resources. A study on adults showed that participants chose the self-advantaging allocation when a fair procedure was optional, even knowing that the other recipient had the power to reject the offer (Bolton et al., 2005). They might have underestimated recipients’ aversion to an unfair procedure (Bolton et al., 2005). Another study on children found 6-year-olds chose a biased procedure to distribute an extra item rather than throw it away (Shaw and Olson, 2014). The application of procedural distribution, whether it is fair or not, might mitigate a proposer’s aversion to unequal outcomes. Due to the uncertainty and controllability of procedural distribution, procedures can be used to maximize one’s benefits while maintaining a fair appearance. In fact, children at 9 were able to use procedures to hide their intentions for self-interest maximization (Shaw et al., 2014). The manipulability of procedural justice perhaps explains why people’s belief in distributive justice rather than procedural justice at an individual level is more predictive of their self-rated health (Lucas et al., 2011). Therefore, procedural justice is not entirely the same as distributional justice in terms of uncertainty and manipulability, suggesting the importance of investigating children’s development in the area of procedural justice.

In this study, we compared children’s preference for the fair procedure when it was set against a disadvantageous procedure (DI) to that when it was set against an advantageous procedure (AI). By checking the difference and how it develops through the early age periods, we can test whether the findings in distributive justice can be applied to procedural distribution. This will contribute to our current understanding of children’s development of fairness norms.

We adopted a procedural choice paradigm similar to the forced choice paradigm used in distributive justice (Fehr et al., 2013; Li et al., 2016; Williams and Moore, 2016). Children aged 3–8 years old were asked to distribute two unequal items (one is big and the other is small) between themselves and another recipient, thus precluding possibilities for an equal distribution. Instead of allocating resources directly, children were asked to choose between two procedures (wheels): one was to provide each child with equal chances to get the bigger reward, and the other was to provide more opportunities in the AI condition, or fewer opportunities in the DI condition, for themselves to get the bigger reward. This allowed us to compare children’s procedural inequity aversion to AI and DI directly. Three age groups were recruited between 3 and 8 years old because inequity aversion to DI and AI have been shown to emerge during this developmental period (Blake and McAuliffe, 2011; Smith et al., 2013; Shaw and Olson, 2014). By examining differences between these age groups, we could examine how the underlying motivations develop during childhood.

We hypothesized that children would show fairness concern in procedural justice context, as consistently shown in distributive justice context. Because children had to overcome their desire for increased chances to win the bigger reward to enforce the fairness norm in the AI condition, we expected a stronger aversion to DI than to AI in younger children. As they grow older, the discrepancy between DI and AI would become smaller.

## Materials and Methods

### Participants

Participants were 96 children, 4-year-olds [*n* = 32, *M*_age_ = 51 months, *SD* = 3.84, *Range* = (43, 56); 19 girls and 13 boys], 6-year-olds (*n* = 32, *M*_age_ = 75 months, *SD* = 3.90, *Range* = (65, 82); 14 girls and 18 boys], 8-year-olds (*n* = 32, *M*_age_ = 103 months, *SD* = 3.63, *Range* = (97, 107); 18 girls and 14 boys]. One participant was excluded from the study because of having difficulty in understanding the protocol. The rest of the participants completed all the trials in the study. Participants were mainly from families with mid- to high- socio-economic status (SES). Among 83% of parents who provided their education experience, 53% of fathers had received undergraduate or higher education, and another 35% received high school education. For the mothers, 53% received undergraduate or higher education, and another 38% received high school education. Parents’ average subjective rating of their SES on a 9-point social ladder measure (Piff et al., 2010), with higher numbers indicating a better placement in comparison to others, was 5.63 (*SD* = 1.53). Participants were recruited from local kindergartens and an elementary school in a northern city in China with agreements to participate in the study obtained from their parents. Our study was approved by the ethics committee of the Institute of Psychology, Chinese Academy of Sciences.

### Materials

#### Wheels

**FIGURE 1:**
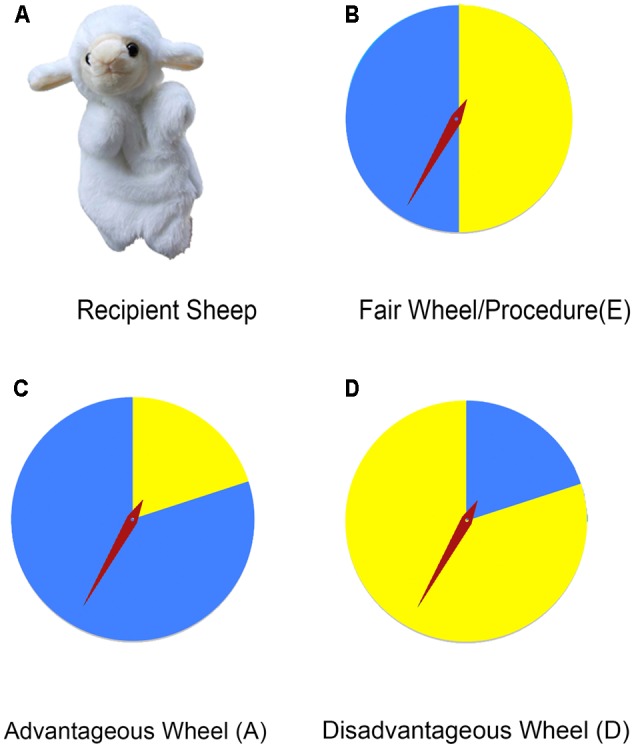
**(A–D)** The recipient sheep puppet and the shape of wheels. If the pointer points to the blue area when the wheel stops in **(B–D)**, the participant will get the bigger reward and the puppet will get the smaller reward. If the pointer points to the yellow area, in contrast, the puppet will get the bigger reward and the smaller reward will go to the participant.

Wheels were used as procedures to allocate resources (see **Figures 1B–D** for the wheels). All the wheels were colored in blue and yellow, but with different proportions. Spin the wheel, and it would rotate until stopping randomly at blue or yellow area. If a wheel stopped at blue area, the bigger reward went to the participant child and the smaller reward went to the other recipient (see **Figure 1A**). Thus, blue was the participant’s lucky color. We put a sheet of blue paper on the participants’ seat to help him/her remember it. There were three wheels: an equal and fair wheel, an advantageous wheel, and a disadvantageous wheel. The fair wheel had the same area of blue and yellow, thus providing equal opportunities for both parties to get the bigger reward. The advantageous wheel had more blue area than yellow (80% of the area covered in blue), thus favoring the participant by offering him/her more chances to get the bigger reward. The disadvantageous wheel, in contrast, had more yellow area (only 20% covered in blue).

#### Rewards

Pairs of fruit erasers were used as rewards to be distributed. Each pair had two erasers with the same shape but different sizes. The bigger eraser was almost three times larger than the smaller one. Different kinds of fruit erasers (such as orange, kiwi, grape, strawberry, watermelon) were used in each trial to keep the reward novel and appealing.

### Procedure

A 3 (age: 4-, 6-, and 8-year-old) × 2 (conditions of inequality: AI and DI) design was adopted in this experiment. Each child participated in all the inequality conditions. Experiments were conducted in a quiet classroom in the kindergartens or schools. Before participants came, the experimenter put a blue sheet of paper on the participant’s seat. There was only one trial in every inequality condition. In each trial, there were two wheels in front of the participant: one was the fair wheel, the other was either the advantageous wheel (in the AI condition) or the disadvantageous one (in the DI condition). The sequence of conditions and the position of the fair wheel (left/right) were counterbalanced.

#### Warm-Up

Participants were introduced to the puppet sheep (manipulated by the experimenter) and the rewards at first. Children were shown two fruit erasers in the same shape but different sizes. They were asked if they liked the erasers (all the participants said “yes” in this experiment), and were told that the sheep liked them as well. Participants were informed that they would allocate the items between him/herself and the sheep.

#### Introduction to the Wheels

Experimenter pointed out that sizes of the erasers were different, and recommended participants to allocate them by the wheels. Then, the experimenter went on to introduce the two wheels sitting in front. The experimenter told the participant, “Look at these wheels. They both have blue and yellow color on it, right? So, spin the wheel [the experimenter acted when she said]. When it stops, the pointer is likely to stop at the yellow zone. [Spin again] It is also possible to stop at the blue zone. See what’s the color of the paper you are sitting on? [the participant said blue] Blue, right? So, blue is your lucky color today! If the wheel stops at blue, you will get the big one, and the sheep will get the small one. If the wheel stops at yellow, you will get the small one, and the sheep will get the bigger one.” “Then the experimenter checked whether participants understood how the two colors were related to the rewards [If a participant failed on the checking answer, the experimenter kept telling the instructions until the participant understood].”

After participants understood the meaning of the colors, the experimenter began introducing the two wheels. She always started with the left one. Take the AI condition and fair wheel on the left as an example, “This wheel has the same area of blue and yellow on it. If you spin this wheel, you and the sheep will have the same amount of opportunities to get the big reward. This wheel has more blue area than yellow. If you choose this, you have a better chance to get the bigger reward, and the sheep has a smaller chance.” The participants were then checked whether they understood the difference between the wheels: “Now, which wheel has more blue/yellow area? Which one has the same amount of blue and yellow?”

#### Test Phase

Finally, participants were asked to choose the wheel, spin it, and got their rewards accordingly. Participants were given 1 for choosing the fair wheel, and 0 for choosing the unfair wheel. All participants succeeded in making a choice.

## Results

**FIGURE 2:**
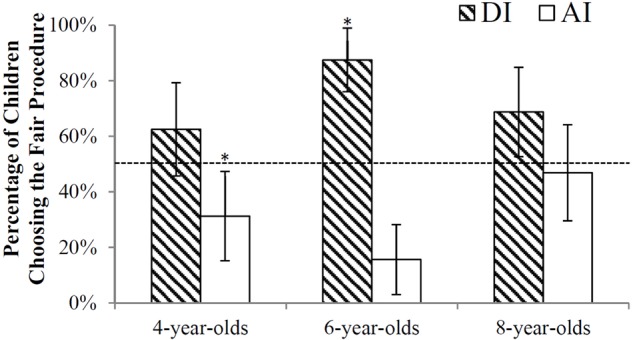
Percentage of children choosing the fair procedure in AI and DI conditions in three age groups. The dash line represents the chance level (50%). ^∗^*p* < 0.05, ^∗∗^*p* < 0.01.

Analyses were conducted in SPSS 19.0. Chi-square tests were run to see whether the spatial position of unfair wheel (Left or Right) or the sequence of conditions would influence children’s choice (see **Figure 2**). Results showed that children’s decisions did not differ as a function of the spatial position of unfair wheels (*p*s > 0.1). The sequence of conditions influenced children’s choices in DI [χ^2^(1) = 11.16, *p* = 0.001, *Cramer’s V* = 0.341], but not in AI [χ^2^(1) = 0.55, *p* > 0.1, *Cramer’s V* = 0.076]. Children who first engaged in AI trials were less likely to choose the fair procedure in DI (57% choosing the fair procedure) than those who first engaged in the DI condition (88% choosing the fair procedure). Further analysis in the three age groups showed that 4-year-olds were strongly influenced by the sequence of conditions [33% vs. 88%; χ^2^(1) = 10.25, *p* = 0.001, *Cramer’s V* = 0.566], whereas for the two older age groups, sequence made no difference (*p*s > 0.1). Moreover, there were no gender differences between children’s procedural choices. Thus, gender was excluded from further analysis.

To analyze whether children randomly made their decisions, binominal tests were run to compare their choices to the chance level. Results showed that 4-year-olds chose randomly between the fair and the unfair procedure in DI (63% choosing the fair procedure, *p* = 0.215). However, their preference for the fair procedure was significantly lower than the chance level in AI (31% choosing the fair procedure, *p* = 0.050). McNemar test showed that 4-year-olds were more likely to choose the fair procedure in DI than in AI (χ^2^(1) = 6.25, *p* = 0.012, 95% CI for odds ratio [1.23, 15.21] (Sahai and Khurshid, 1995)). Six-year-olds showed a clear preference for the fair procedure in DI (88% choosing the fair procedure, *p* < 0.001), but a preference for the advantageous procedure in AI (16% choosing the fair procedure, *p* < 0.001). McNemar test showed that 6-year-olds were more likely to choose the fair procedure in DI than in AI (χ^2^(1) = 21.16, *p* < 0.001, 95% CI for odds ratio [3.24, 177.41]). Eight-year-olds also preferred the fair procedure beyond the chance level in DI (69% choosing the fair procedure, *p* = 0.050), but they chose randomly between the wheels in AI (47% choosing the fair procedure, *p* = 0.860). McNemar test showed no difference in children’s preference for the fair procedure between DI and AI conditions (χ^2^(1) = 2.33, *p* = 0.127, 95% CI for odds ratio [0.81, 4.95]). Results suggested that children at 4 and 6 had a greater preference for the fair procedure in DI than that in AI. For 8-year-old children, however, their preference for the fair procedure was not significantly different between the conditions.

Chi-square tests were run to compare children’s choices of the fair procedure across different age groups. Results revealed a greater number of 6-year-old children choosing the fair procedure in DI than that of 4-year-olds [Fisher’s Exact χ^2^(1) = 5.33, *p* = 0.021, *Cramer’s V* = 0.289] or 8-year-olds [Fisher’s Exact χ^2^(1) = 3.29, *p* = 0.070, *Cramer’s V* = 0.227], and fewer 6-year-old children choosing the fair procedure in AI than 8-year-olds [χ^2^(1) = 7.27, *p* = 0.014, *Cramer’s V* = 0.337], with no significant difference in other comparisons. These results suggested that 6-year-olds were more likely to choose the fair procedure in DI, but the advantageous procedure in AI compared to 8-year-olds.

**FIGURE 3:**
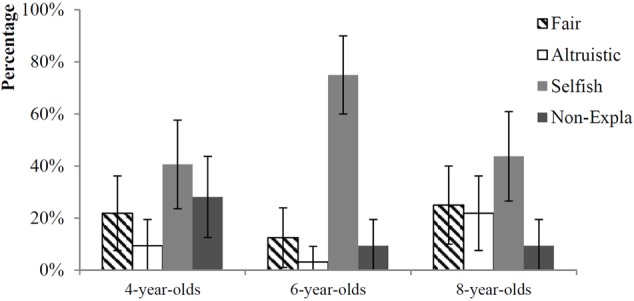
Percentage of children who were fair, altruistic, selfish, or non-explainable in three age-groups. Fair refers to the children choosing the fair procedures in both conditions. Altruistic indicates the children always choosing the other-profit maximizing procedure (D wheel in DI and E wheel in AI). Selfish indicates the children always choosing the self-profit maximizing procedure (E wheel in DI and A wheel in AI). Non-explainable indicates the children who chose D in DI but A in AI.

In the following analysis, children in each age group were divided into four categories according to their choices in both conditions: fair (choosing the fair procedure in both conditions), altruistic (choosing the disadvantageous procedure in DI and the fair procedure in AI), selfish (choosing the fair procedure in DI and the advantageous procedure in AI), and non-explainable (choosing the disadvantageous procedure in DI and the advantageous procedure in AI, see **Figure 3**). Chi-square tests were run to compare the difference across age groups. The only significant difference found between age groups was the number of selfish children [χ^2^(2) = 9.29, *p* = 0.010, *Cramer’s V* = 0.311]. More children at 6 chose selfishly (75%) than children at 4 [41%, χ^2^(1) = 7.75, *p* = 0.005, *Cramer’s V* = 0.348] or at 8 [44%, χ^2^(1) = 6.48, *p* = 0.011, *Cramer’s V* = 0.318]. Other comparisons between age groups were not significant [altruistic: Fisher’s Exact χ^2^(2) = 5.28, *p* = 0.074, *Cramer’s V* = 0.245; non-explainable: χ^2^(2) = 5.69, *p* = 0.058, *Cramer’s V* = 0.243; fair: χ^2^(2) = 1.71, *p* = 0.426, *Cramer’s V* = 0.133]. Results indicated that 6-year-olds were more likely to choose the procedure that would maximize their self-interest than 4-year-olds and 8-year-olds.

## Discussion

This study investigated children’s procedural inequity aversion under AI and DI conditions. Results showed an age-related difference. Children in 4- and 6-year-olds showed a stronger inequity aversion to DI than to AI: they cared more about fairness when they would be disadvantaged by, than when they would benefit from, the inequity conditions. In contrast, the difference between DI and AI was not significant in 8-year-olds, suggesting an emergence of other-oriented concern as children get older. Moreover, 6-year-olds were more likely to choose the procedures that could maximize their chances to win the better reward than the other two age groups.

Our results suggest that children at 4 were able to tell the difference between the wheels and made decisions that served to their personal interest. They would abandon the fair procedure in AI, and their likelihood to choose the fair procedure was much greater in DI. This is also the case for 6-year-olds. The differentiation between aversion to DI and to AI is consistent with results found in distributive justice (Fehr et al., 2008; Blake and McAuliffe, 2011). Different amount of cognitive control needed to choose the fair procedure may help explain the findings (Blake and McAuliffe, 2011; Fliessbach et al., 2012). Age-related increase in cognitive control has been found to explain why children strategically offer more to others (Steinbeis et al., 2012). In the present study, the fair procedure in DI was to the participants’ advantage, providing relatively more opportunities to obtain the bigger reward in addition to maintaining fairness. In the AI condition, however, the fair procedure provided less chance of winning the bigger reward compared to the alternative, advantageous procedure. As a result, children were more likely to experience the conflict of maximizing one’s self-interest and being fair. Younger children might not develop sufficient cognitive control to combat the desire for bigger rewards, as suggested in other studies (Smith et al., 2013). In addition, 4-year-olds were more likely to choose the disadvantaged procedure if they engaged in the AI condition first.

As mentioned earlier, cognitive ability alone may not fully explain the results. If cognitive control is the only reason for the differentiation, aversion to DI and AI should develop at the same rate as children’s cognitive control increases over the years (Diamond, 2013). However, in this study, children’s aversion to DI reached its peak at around 6 years old, whereas aversion to AI started to appear at the age of 8. The results indicated that inequity aversion might be driven by other motivations at an earlier age. One of them could be social comparison: children’s preference for the fair procedure in DI was perhaps not out of fairness concern, but to prevent others from getting more resources and winning a relative advantage (Blake et al., 2014). This is consistent with Sheskin et al. (2014) who found that children at 5–6 cared so much about social comparison that they would take a cost to get more than others. As children grew older, they might gradually attach more weight to social norms than social comparison (Steinbeis and Singer, 2013; Sheskin et al., 2014). Another possibility is that children cared only about their own benefit, trying to maximize their chances to win without considering the other recipient’s interest at all. Both of these assumptions indicated that children’s inequity aversion to DI in the early years might not be due to fairness concern, but to maximize their relative self-interest.

The rise of aversion to AI, and the smaller gap between the two kinds of inequity aversions in the oldest age group suggests a possible transition from self-interest maximization to other-oriented motivations (e.g., fairness concern and altruism). Children may become more aware of fairness norms with age, as they may outgrow the selfish inclinations and value fairness above personal gain (Blake and McAuliffe, 2011). In addition, nearly one-third of the 8-year-olds chose the disadvantageous rather than the fair procedure in DI. This was mainly due to an increasing number of children choosing altruistically, that is, choosing the disadvantaged procedure in DI and the equal procedure in AI. This is consistent with Shaw et al. (2016) who found older children were more likely to choose unequal options that disadvantaged themselves to show their generosity and kindness than 4- to 6- year-olds. In addition, Chinese children were educated not only to be fair but also to be altruistic (Chen et al., 2013). Older children in our study were exposed to more moral education than the younger children. This may explain why they chose disadvantageous inequity. Children at 8 cared more than just self-interest. Instead, they may want to build a good reputation by being fairly or even altruistically.

Although 8-year-old children had a larger tendency to choose the fair procedure in AI, their choices were not different from the chance level. This is different from results in distributive justice indicating that children at 8 are willing to sacrifice some self-interest to make the distribution more fair (Blake and McAuliffe, 2011; McAuliffe et al., 2013, 2014; Smith et al., 2013; Shaw et al., 2016; Williams and Moore, 2016). This result is also different from results found in procedural justice suggesting that children at 8 have developed a sense of procedural justice (Shaw and Olson, 2014) and are able to reject outcomes out of unfair procedures (Grocke et al., 2015). There are two explanations for this discrepancy. First, our study is different from previous studies on procedural justice in that children chose actively between the procedures with their self-interest involved. Children may concern less about the fairness of outcomes when choosing procedures to allocate resources. Previous studies have shown that people would choose the self-advantaging distribution when a fair procedure was optional (Bolton et al., 2005). Six-year-olds would choose a biased procedure to determine the distribution of an extra item rather than throw the item away (Shaw and Olson, 2014). The application of procedural distribution, whether it is fair or not, might mitigate children’s aversion to unequal outcomes. Due to the uncertainty and controllability of procedural distribution, procedures can be used to maximize one’s benefits while maintaining a fair appearance (Shaw et al., 2014). Using procedures, children might feel less urged to act fairly. Another explanation concerns the social context. Hand puppet used in this study might reduce social influence, especially among older children. The attributes of the recipients were essential in AI condition, as children’s aversion to AI would disappear in non-social settings (McAuliffe et al., 2013). Specific reasons for children’s relative low performance of advantageous inequity aversion in the present study remain unclear. Both the application of procedures and the characteristic of recipients may affect children’s inequity aversion. Further research is needed to test these conjectures.

### Limitations

One limitation of the present study was use of hand puppet to represent recipients. This setup might decrease children’s willingness to enforce fairness norms due to less social pressure. We used hand puppet to make the procedure more consistent among participants and make the results more comparable to other studies (Sheskin et al., 2014; Williams and Moore, 2016). It is also worth noting that even in such circumstances, children were hesitant to pursue their self-interest. Further studies should be conducted to investigate the influence of recipient characteristics on children’s fairness consideration (e.g., Yu et al., 2016). Another limitation was that we recruited different age groups of children but did not follow the same groups of children from 3 to 8 years old. Future studies can adopt longitudinal designs to reveal developmental trajectories of children’s fairness concern in procedural justice context.

## Conclusion

When distributing uneven resources by choosing between a fair procedure and an unfair procedure to spin, children aged 4 and 6 were more likely to choose the fair procedure when the alternative procedure implied a larger possibility of disadvantaging them than maximizing their chances to win. The discrepancy between aversion to DI and to AI reduced in the 8-year-olds, however, as their aversion to the advantageous procedure increased. The results indicate that children in the early years are generally driven by self-interest and lack sufficient cognitive control to inhibit this tendency. At the age of 8, however, they start to show more concern for fairness.

## Ethics Statement

This study was carried out in accordance with the ethics committee of the Institute of Psychology, Chinese Academy of Sciences with written informed consent from all subjects’ parents. All subjects gave written informed consent in accordance with the Declaration of Helsinki. The protocol was approved by the ethics committee of the Institute of Psychology.

## Author Contributions

XQ and LZ designed the experiment. XQ collected the data. XQ and NC analyzed the data. XQ, JY, TL, NC, and LZ wrote the manuscript.

## Conflict of Interest Statement

The authors declare that the research was conducted in the absence of any commercial or financial relationships that could be construed as a potential conflict of interest.
